# Clinical supervision effectiveness in NHS nursing, medical and allied health professionals: Exploring interaction with workplace factors, supervision factors and burnout

**DOI:** 10.1111/jep.14149

**Published:** 2024-10-22

**Authors:** Emma Sellers, Sarah Craven‐Staines, Claire Vaughan

**Affiliations:** ^1^ Department of Clinical and Health Psychology Leeds Teaching Hospitals NHS Foundation Trust Leeds UK; ^2^ Department of Psychology Teesside University Middlesbrough UK; ^3^ Durham Tees Valley ALD Tees, Esk, and Wear Valleys NHS Foundation Trust Darlington UK

**Keywords:** burnout, clinical supervision effectiveness

## Abstract

**Objectives:**

The study aimed to (1) determine if a variety of workplace and supervision factors predict clinical supervision effectiveness; and (2) establish if clinical supervision effectiveness predicts burnout, amongst a variety of mental health staff (medical, allied health, and nursing staff).

**Design:**

The study adopted a multicentre cross‐sectional online survey design.

**Methods:**

Participants included 204 mental health staff (89 allied health staff, 81 nursing staff, and 34 medical staff). The Manchester Clinical Supervision Scale (MCSS‐26) was used to measure clinical supervision effectiveness, and the Maslach Burnout Inventory (MBI‐HSS) was used to measure burnout. Linear regression analyses and multiple regression analyses were conducted.

**Results:**

The main findings suggested that supervision frequency, supervision duration, choice of supervisor, workplace setting, and supervisee profession, were all significant predictors of clinical supervision effectiveness. Additionally, clinical supervision effectiveness was a significant negative predictor of burnout.

**Conclusions:**

Workplace and supervision factors should be considered in supervision practice across professional groups. Policies need to promote effective clinical supervision practice.

## 1 INTRODUCTION

Clinical supervision is defined as a safe and confidential space for staff to discuss their work and responses to it, with a focus on supporting reflective practice and personal and professional development.^1^ It is widely implemented across healthcare services owing to its positive impact on patients and organisations, including greater staff retention,^2^ and reduced mortality rate of patients.^3^, ^4^ The National Health Service (NHS) has a responsibility to ensure that clinical staff have access to effective clinical supervision.^5^ Effective clinical supervision must meet the supervisees needs, providing education, support, and guidance for professional practice.^6^, ^7^ Proctor's^8^ model outlines three supervision functions: to be Normative, Formative, and Restorative. Effective clinical supervision may provide support in any or all three of these domains.^8^ Antecedents of effective clinical supervision therefore need to be identified.^9^

We conducted a systematic review to explore factors influencing clinical supervision effectiveness. The review included 10 studies.^4^, ^10^, ^11^, ^12^, ^13^, ^14^, ^15^, ^16^, ^17^, ^18^ These studies involved a total of 2114 participants, and identified a large variety of supervision and workplace factors explored in previous research, several of which were identified as influential on clinical supervision effectiveness. The review highlighted that clinical supervision effectiveness was significantly influenced by supervision frequency in the majority of studies^4^, ^11^, ^13^, ^14^ but not all.^16^ It was also significantly positively associated with supervision duration in several studies.^4^, ^11^, ^14^, ^15^ Hyrkäs et al.,^13^ however, suggested that participants receiving 45‐min sessions experienced more effective clinical supervision compared to longer sessions; notably they did not provide response options for supervision with less than 45‐min duration. Snowdon et al.^16^ and Hyrkäs et al.^13^ found no significant relationships between supervision duration and clinical supervision effectiveness. Having a choice of supervisor was consistently found to be associated with more effective clinical supervision than those with allocated supervisors.^11^, ^13^, ^14^, ^15^, ^17^ Supervisee profession was also consistently identified as having an association with clinical supervision effectiveness^10^, ^12^, ^13^, ^17^; however, due to homogeneous samples (e.g., not comparing across a variety of professional groups) generalisability is problematic. Only one study looked at delivery method;^18^ no significant differences were found between in person and audio only supervision. This review highlighted several variables which may be important to consider when implementing clinical supervision. Nevertheless, definite conclusions cannot be drawn due to the limited research in this field, the limited number of studies exploring each variable, and research predominately being conducted solely with individual professional groups.

### 1.1 Clinical supervision and burnout

Burnout is considered a by‐product of chronic occupational stress^19^ which involves the sum of three components: emotional exhaustion (EE); depersonalisation (DE); and reduced personal accomplishment (PA).^20^ The Job Demands Resources Model^21^ proposes that job resources (such as clinical supervision) can act as a buffer to the impact of job demands, by reducing stress and burnout.^22^, ^23^

EE has been found to be negatively associated with some clinical supervision effectiveness scores in some research,^7^, ^12^, ^13^, ^24^, ^25^ but not in others^10^, ^26^ DE has also been found to be negatively associated with some clinical supervision effectiveness scores.^7^, ^10^, ^12^, ^24^, ^26^ Hyrkäs et al.^13^ found no associations between DE and clinical supervision effectiveness. PA has been found to have a positive association with clinical supervision effectiveness.^7^, ^24^, ^25^, ^26^ Yet, some studies found no association in relation to PA.^10^, ^12^, ^24^ Martin et al.'s^2^ systematic review included a synthesis of five of these quantitative studies (*n* = 1046)^7^, ^10^, ^12^, ^24^, ^26^ and concluded that clinical supervision effectiveness may be significantly negatively associated with EE and DE, but not with a lack of PA. Previous research is limited, inconclusive, and the majority of studies in this area have been conducted solely with nursing staff, highlighting the need for further research.

As the current study recruited participants in between 2021 and 2022 it was important to consider the Covid‐19 pandemic and its impact on clinical supervision provision. Increased work demands, stress and pressure on healthcare staff during the pandemic, is likely to have taken time away from support systems such as clinical supervision.^27^

### 1.2 Aims and hypotheses

This study primarily aimed to explore which workplace and clinical supervision factors influence the effectiveness of clinical supervision in a range of mental health professionals. Details of the primary predictor variables can be found in Table 1. The secondary aim of this research was to determine the relationship between the effectiveness of clinical supervision and burnout. Hypotheses were based upon findings from the aforementioned systematic review, and Martin et al.'s systematic review.^2^

**Table 1:** Details of the primary predictor variables explored in the current study.

Type of variable	Predictor variables (categorical response options)
Workplace factors	Workplace setting *(inpatient/community)* Clinical Specialty *(adult/child and adolescent/older adults/learning disability/forensic)* Shift pattern *(day shifts/night shifts/a mixture of day and night shifts)*
Supervisee factors	Working hours *(full time/part time)* Supervisee profession *(allied health/nursing/medical)* Supervisee sex *(male/female/other)* Supervisee age Duration in current role
Clinical supervision infrastructure factors	Frequency of supervision Duration of supervision Duration of supervisory relationship Choice of supervisor *(chosen/allocated)* Supervision delivery method *(face‐to‐face/virtual)*
Clinical supervision infrastructure factors due to Covid‐19	Change in frequency of supervision during Covid‐19 *(decreased/stayed the same/increased)* Change in duration of supervision during Covid‐19 *(decreased/stayed the same/increased)* Change of supervision delivery method during Covid‐19 *(decreased/stayed the same/increased)*
Clinical supervisor factors	Supervisor sex *(male/female/other)*

Hypothesis 1 The following factors would explain a significant proportion of the variance in clinical supervision effectiveness:

a) Supervisee sex (being female shall be associated with greater supervision effectiveness);
b) Frequency of supervision (more frequent supervision sessions will be associated with greater supervision effectiveness);
c) Duration of supervision (having longer supervision sessions will be associated with greater supervision effectiveness);
d) Choice of supervisor (having choice of supervisor shall be associated with greater supervision effectiveness).

Hypothesis 2 Nondirectional hypotheses were made for the following variables:

a) Workplace setting;
b) Shift pattern;
c) Clinical specialty;
d) Supervisee profession;
e) Supervisee age;
f) Working hours;
g) Duration in role;
h) Duration of supervisory relationship;
i) Supervision delivery method;
j) Change in frequency of supervision during Covid‐19;
k) Change in duration of supervision during Covid‐19;
l) Change of supervision delivery method during Covid‐19;
m) Supervisor sex.

Hypothesis 3 Clinical supervision effectiveness would negatively explain a significant proportion of the variance in burnout scores.

## 2 METHOD

### 2.1 Inclusion criteria

Participants were included if they were: a clinical member of staff (delivering direct clinical care with patients); and were a paid employee of one of the two recruiting North East mental health trusts.

### 2.2 Setting

Participants were recruited through Tees, Esk, and Wear Valley NHS Foundation Trust (TEWV) and Leeds and York Partnership NHS Foundation Trust (LYPFT). There are not an all‐encompassing models, guidelines or policies for supervision used within these sites or across professional disciplines making it likely that the provision of supervision is variable.

### 2.3 Design

This multi‐site research study was quantitative in nature, and adopted a cross‐sectional, online survey design. All participants completed a 10‐min online form comprising four measures. Three clinicians were involved in the development of the research design.

### 2.4 Measures

1) *Clinical Supervision Factors Questionnaire*. This 13 item purpose built, non‐validated questionnaire collected information such as supervision duration and frequency.
2) *Manchester Clinical Supervision Scale (MCSS‐26)*.^28^ This 26‐item questionnaire measures the effectiveness of clinical supervision from the supervisee's perspective. The questionnaire measures six factors: Trust and Rapport (TR); Supervisor Advice and Support (SAS); Improved Care and Skills (IMP); Importance and Value of clinical supervision (IMV); Finding Time (FT); and Reflection (REF). These subscales map onto the Proctor's Model^8^ of clinical supervision.^29^ Higher MCSS‐26 total scores indicate greater clinical supervision effectiveness; a score of ≥73 suggests effective clinical supervision.^29^
3) *The Maslach Burnout Inventory (MBI‐HSS)*.^20^ This 22‐item questionnaire measures burnout. The MBI‐HSS measures three components of burnout: Emotional Exhaustion (EE), Depersonalisation (DE), and low sense of Personal Accomplishment (PA). High levels of burnout are characterised by EE scores of ≥27, DE scores of ≥13, and PA scores of ≤31. The author recommends analysing burnout scores, not categories.^30^ Scores must be significant across all three domains to significantly predict burnout.^31^
4) *The Workplace and Demographic Factors Questionnaire*. This nine item purpose built, nonvalidated questionnaire collected information such as age and profession.

Both the MCSS‐26 and the MBI‐HSS are widely used and internationally validated, with extensive data on their effective psychometric properties.^32^, ^33^

### 2.5 Procedure

Participants were invited via NHS email, electronic staff newsletter, and staff intranet pages. The study invitation contained basic study information and a link to the participant information sheet. Participants had to confirm eligibility and consent to participate to proceed. If participants did not fully complete the questionnaire, their data was not collected. Participants had 2 weeks from the date of participation to withdraw their data; however, no withdrawal requests were received.

### 2.6 Ethics

Ethical approval was granted from the hosting University, Health Research Authority, and Research and Development departments from each Trust, before data collection. Informed consent was granted from participants. Participants were informed of where to access support if needed. Data was anonymous, treated confidentially, and stored securely.

### 2.7 Data analysis

All statistical analyses were conducted using SPSS Statistical Analysis Software. Statistical results are reported to two decimal places, except for *p* values, in keeping with American Psychological Association (APA) guidance (APA, 2020). Significance tests were two‐tailed, and *p* < 0.05 was the definition of significance. Effect sizes were calculated using Pearson's *r* where appropriate. A post hoc power calculation using G*Power software suggested that 99% power was attained with a sample size of 204 in this study, based on a multiple regression‐based analysis with 24 predictor variables (accounting for categorical dummy variables).

To address the primary research aim, multiple regression analyses were conducted to assess the impact a contribution of workplace and supervision factors had on clinical supervision effectiveness (MCSS‐26 total score). To address the secondary aim, the MCSS‐26 total score was entered in a linear regression with the three subscales of the MBI‐HSS.

Before conducting all analyses, relevant assumptions were assessed. Regression outputs were assessed for unusual cases, independence of observations, homoscedasticity of residuals, linearity, and multicollinearity.^34^ Categorical variables were dummy coded to input into the regression models. Linearity between the predictor and outcome variables were assessed by partial regression plots. Homoscedasticity of residuals and collective linearity were assessed via visual inspection of scatterplots of standardised residuals plotted against unstandardised predicted values.

## 3 RESULTS

### 3.1 Participants

Participants included 204 clinical members of staff, who received clinical supervision. Initially, 212 participants completed the questionnaire, though eight participants reported not receiving clinical supervision therefore were excluded from statistical analysis. 59 people did not complete and submit responses, suggesting a completion rate of 78%.

The sample included 50 males (25%) and 154 females (75%) from a variety of professional backgrounds including 89 (44%) allied health staff, 81 (40%) nursing staff, and 34 (17%) medical staff. 123 participants were recruited through TEWV (60%) and 81 participants were recruited through LYPFT (40%).

### 3.2 Descriptive statistics

Sixty‐five percent of the sample received effective clinical supervision (*M* = 76.81; SD = 9.43), and in terms of burnout 28% of the sample experienced high levels of EE (*M* = 22.24; SD = 6.59), 3% were experiencing high levels of DE (*M* = 6.97; SD = 2.93), and 31% were experiencing low levels of PA (*M* = 34.47; SD = 6.06).

### 3.3 Factors influencing clinical supervision effectiveness

This multiple regression model significantly predicted the MCSS‐26 total score, *F*(21, 182) = 12.39, *p* < 0.001, explaining 59% of the variance (*R* ^2^ = 0.59). Following this analysis, the five variables found to be significant predictors were re‐entered into a separate multiple regression, with the aim of producing a more parsimonious model. This multiple regression model statistically significantly predicted the MCSS‐26 total score, *F*(5, 198) = 48.95, *p* < 0.001 explaining 55% of the variance (*R* ^2^ = 0.55); a large effect size according to Cohen.^35^ The five variables found to significantly predict clinical supervision effectiveness were workplace setting (*p* < 0.01), supervisee profession (*p* < 0.001), choice of supervisor (*p* < 0.001), supervision frequency (*p* < 0.001), and supervision duration (*p* < 0.001). In terms of supervisee profession, being an allied health professional compared to nursing staff, was a significant predictor of higher clinical supervision effectiveness; differences with medical staff, however, were not significant. Table 2 presents these variables' means and standard deviations.

**Table 2:** Means and standard deviations of MCSS‐26 total score by significant workplace and supervision factors.

Significant workplace and supervision factors	*n*	MCSS‐26 Score	
		*M*	SD
Workplace setting			
Community	135	79.01	8.90
Inpatient	69	72.49	8.95
Supervisee profession			
Allied Health	89	79.91	9.11
Nursing	81	73.52	8.88
Medical	34	76.74	9.13
Choice of supervisor			
Chosen	87	82.05	7.62
Allocated	117	72.91	8.77
Supervision frequency			
Once a week	25	84.68	6.65
Once every 2–3 weeks	29	82.21	5.80
Once every 1–2 months	111	77.02	8.88
Once every 3–4 months	21	68.81	6.25
Once every 5–6 months	10	68.10	4.68
Once every 7–8 months	4	62.00	2.83
Once every 9–12 months	4	61.25	3.78
Supervision duration			
>60 min	28	85.30	6.60
46–60 min	133	77.40	8.12
30–45 min	27	71.40	5.81
<30 min	16	66.10	4.95

Abbreviations: *M*, mean; SD, standard deviation.

Eleven factors did not significantly predict the MCSS‐26 total score: supervisee sex, clinical specialty, shift pattern, working hours, duration in role, supervisee age, duration of supervisory relationship, change in duration of supervision during Covid‐19, change in frequency of supervision during Covid‐19, change of supervision delivery method during Covid‐19, and supervisor sex.

Two factors were not included in the analysis. Supervisor profession was not included due to its high correlation with supervisee profession, and supervision format was not included due to the small sample size of participants receiving group supervision (*n* = 3).

### 3.4 Clinical supervision effectiveness and its impact on burnout

To address the secondary aim of this research, three linear regressions were conducted to assess the contribution clinical supervision effectiveness (MCSS‐26 total score) had on each component of burnout (EE, DE, PA) as measured by the MBI‐HSS.

Clinical supervision effectiveness negatively significantly predicted EE, *F*(1, 202) = 186.09 *p* < 0.001, adjusted *R* ^2^ = 48%; a substantial effect size.^35^ Clinical supervision effectiveness negatively significantly predicted DE, *F*(1, 202) = 145.30 *p* < 0.001, adjusted *R* ^2^ = 42%; a medium effect size.^35^ Clinical supervision effectiveness significantly predicted reduced PA, *F*(1, 202) = 81.94 *p* < 0.001, adjusted *R* ^2^ = 29%; a small effect size.^35^ As findings were significant across all three MBI‐HSS domains, it can be interpreted that clinical supervision effectiveness negatively explains a significant proportion of burnout variance.

## 4 DISCUSSION

The primary aim of this study was to explore which workplace and clinical supervision factors influence the effectiveness of clinical supervision.

### 4.1 Hypothesis 1

Findings partially supported hypothesis 1, as supervision frequency, supervision duration and choice of supervisor all were significant predictors of clinical supervision effectiveness.

#### 4.1.1 Supervision frequency

Receiving more frequent supervision was predictive of higher perceptions of clinical supervision effectiveness; consistent with previous literature.^4^, ^11^, ^13^, ^14^ Findings highlighted that for clinical supervision to be deemed effective, supervision needed to be at least every 1–2 months; supervision less frequent was not found to be effective. Edwards et al.^11^ proposed that the optimal supervision frequency was at least once a month. Having frequent supervision is likely to create continuity, and consistency of support from the supervisor, facilitating a more trusting relationship.^11^

#### 4.1.2 Supervision duration

Receiving longer supervision sessions predicted higher perceptions of clinical supervision effectiveness. This supports previous research.^4^, ^11^, ^15^ Current findings highlighted that for clinical supervision to be effective, supervision needed to be at least 46–60 min; shorter sessions were not found to be effective. It is likely that longer sessions allow more opportunities for more in depth reflection and feedback, which are key functions of supervision.^8^

#### 4.1.3 Choice of supervisor

Having choice of supervisor was found to be the strongest predictor of clinical supervision effectiveness compared to allocation, supporting previous findings.^11^, ^15^ This is unsurprising, as allowing supervisees to choose their clinical supervisor is recommended practice as it facilitates the development of strong working alliances.^11^

### 4.2 Hypothesis 2

Linked to the primary aim of the research, hypothesis 2 was nondirectional in nature. Two variables were significant predictors of clinical supervision effectiveness (workplace setting and supervisee profession).

#### 4.2.1 Workplace setting

Working in community services compared to inpatient services, predicted higher perceptions of clinical supervision effectiveness. Previous research found no significant difference in work setting; however, this was comparing acute settings versus subacute in physical health services in Australia with physiotherapists.^16^ Recently, Snowdon et al.^36^ have suggested that clinical supervision is more effective in private practice for allied health staff, compared to those working in hospital, community, and education settings. To our knowledge this is the first study exploring the difference between inpatient and community mental health services. Further research is required to understand clinical supervision provision and experiences within inpatient services. Replication research should be prioritised initially.

#### 4.2.2 Supervisee profession

Being an allied health professional predicted higher perceptions of clinical supervision effectiveness compared to nursing staff. Significant differences were not found between medical staff and other professions. These findings add to the existing literature in this area, as previous studies had homogeneous professional samples, which were predominantly comparing different types of nursing staff^10^, ^12^, ^13^; or different types of allied health staff.^4^ Recently, Snowdon et al.^36^ identified significant differences between allied health professionals; with psychologists having more effective clinical supervision than dieticians and allied health assistants. Medical staff remain under‐researched in terms of clinical supervision effectiveness and quality. The current research tried to address this gap in the literature, but they still made up a small part of the recruited sample (*n* = 34, 17%). This could be because medical staff make up a smaller proportion of the mental health workforce, but they still remain an under‐researched population.

### 4.3 Hypothesis 3

Hypothesis 3 aimed to determine the relationship between the effectiveness of clinical supervision and burnout. Results identified that clinical supervision effectiveness is a significant predictor of burnout.

Clinical supervision effectiveness negatively predicted EE, supporting some previous findings.^12^, ^13^, ^24^, ^25^ Two previous studies however, found no association between clinical supervision effectiveness and EE.^10^, ^26^ Disparities between the current results and these previous findings may be due to the different staffing populations studied; Berry and Robertson^10^ and Hyrkäs^26^ research was conducted solely with nursing staff. Given that this is the only study that has been conducted in the last 5 years, and in light of Covid‐19, experiences of emotional exhaustion and supervision practice may have changed.

Clinical supervision effectiveness negatively predicted DE, supporting findings from previous literature.^10^, ^12^, ^24^, ^25^, ^26^

Clinical supervision effectiveness positively predicted PA supporting findings from previous research^13^, ^25^, ^26^ This suggests that when clinical supervision is perceived to be effective it can act as a protective factor against a reduced sense of personal accomplishment, across staffing professions.

### 4.4 Limitations

Self‐report measures are prone to social desirability bias.^37^ The cross‐sectional nature of this study means causality cannot be ascertained, and variables were not controlled for. Findings represent a snapshot of participants experiences; experiences of supervision are likely to fluctuate. The response rate was likely impacted by workplace pressures as recruitment occurred during Covid‐19 meaning clinical roles were likely prioritised over partaking in research.

The chosen workplace and supervision variables only accounted for 55% of the variation in clinical supervision effectiveness. Clinical supervision effectiveness only accounted for 48% of the variation in EE; 42% of the variation in DE; and 29% of the variation in PA. Therefore, other factors that significantly affect clinical supervision effectiveness and burnout were not accounted for and require further research. Due to the complex nature of these topic areas, there are likely a multitude of factors at play. Other factors may include, but are not limited to, supervision model, and other sources of support within and outside the workplace.

To explore differences, participants were grouped into allied health, medical, and nursing professions; this is seen within the structure of clinical directorates within the NHS. Although it is recognised that therapy‐based professions such as psychologists are not allied health professionals, within this study, therapy‐based professionals were grouped within allied health professionals, as this is how the professionals were grouped within clinical directorates in the recruiting NHS trusts. Grouping psychologists within allied health professionals may be problematic as differences in supervision effectiveness have been found.^36^

### 4.5 Strengths

This study explored several workplace and supervision factors in relation to clinical supervision effectiveness. The impact of professional group on clinical supervision effectiveness was considered, across a more heterogeneous sample than previous research. The study being multisite in design, having a large sample size (*n* = 204), and having a more heterogeneous sample of mental health professionals increases the generalisability of the findings, and adds to the limited literature conducted in England. This study additionally considered the context in which the research was being conducted in, namely Covid‐19. By investigating changes to clinical supervision practice during Covid‐19, it allowed for exploration of this possible impact on clinical supervision effectiveness, something which has not been done in the existing literature to date.

### 4.6 Implications for practice

Clinical supervision policies should be informed by and reflect the evidence base, which has been strengthened by this study's findings. Supervisees should be given a choice of their supervisor and receive supervision for at least of 46–60 min once every 1–2 months. When considering the optimal supervision session duration and frequency, having a ‘one size fits all’ approach may not be the best practice. Those with more complex cases may require longer or more frequent clinical supervision sessions. Staff having choice over their supervisor is particularly important, to promote a safer supervisory space. Logistically having this choice may not always be possible due to limited staffing or organisational structures; where this is not possible opportunities to review supervision experience should be embedded in practice and policy. Clinical supervision should be a protected clinical activity, where it is embedded into standard working practice. This may involve scheduling supervision sessions, and ensuring workloads are manageable to allow adequate time for and frequency of supervision. Time attending supervision, away from direct clinical duties, should be perceived as an intervention to support the safety and quality of clinical practice and continued sustainability of the workforce. It is essential that a culture of valuing supervision is built into clinical practice for healthcare staff, potentially this is even more essential for inpatient nursing staff. Policies should aim to promote a safe supervisory environment, which is tailored to supervisees individual needs. Further workforce training may be required around effective clinical supervision practice.

Although clinical supervision policies exist across professions, they may not be implemented due to assumptions about what clinical supervision looks like in practice. Therefore, following a review of the policies, clinical supervision practice needs to be reviewed within teams through service evaluation, to highlight additional areas for intervention.

### 4.7 Future research

Due to the multifaceted nature of clinical supervision, its effectiveness is likely influenced by a complex interaction of workplace and supervision variables, all of which were not accounted for in the current study and require further exploration. Particularly, the influence of supervisee ethnicity should be explored, as this has not been captured within previous research, or the current study.

Findings highlighted that effective supervision practice could be learnt from community services, and that inpatient services are a potential area for development; clinical supervision in differing workplace settings requires further exploration.

### 4.8 Conclusion

Choice of supervisor, supervision frequency, supervision duration, workplace setting, and supervisee profession were all factors found to impact clinical supervision effectiveness across a heterogeneous group of mental health professionals. Clinical supervision effectiveness significantly negatively predicted burnout, highlighting the importance of clinical supervision in practice for all clinical staff members.

## CONFLICT OF INTEREST STATEMENT

The authors declare no conflict of interest.

## Data Availability

The data that support the findings of this study are available from the corresponding author upon reasonable request.
